# Prevalence and Associated Factors of Depression among Admitted Adult Patients in Surgical and Medical Wards of Saint Paul's Hospital Millennium Medical College, Addis Ababa, Ethiopia

**DOI:** 10.1155/2021/8874834

**Published:** 2021-01-31

**Authors:** Merga Siyoum, Getachew Assfaw, Henok Yitbark, Getachew Tesfaw

**Affiliations:** ^1^Research and Training Department, Amanuel Mental Specialized Hospital, Addis Ababa, Ethiopia; ^2^Department of Psychiatry, College of Medicine and Health Science, University of Gondar, Gondar, Ethiopia

## Abstract

**Background:**

Depression is a leading cause of major public health problems globally, and its prevalence has been increasing, particularly in low- and middle-income countries including our country. Therefore, this study is aimed at exploring depression symptoms and their determinants among admitted medical and surgical patients which is important to get optimal care for patients.

**Methods:**

An institution-based cross-sectional study was conducted from May to June 2019, on adults' medical and surgical admitted patients at Saint Paul's Hospital Millennium Medical College, Addis Ababa, Ethiopia. The systematic random sampling technique was used to get a total of 590 samples. The standardized hospital anxiety and depression scale (HADS) was used to assess individual depression symptoms. We computed the bivariate and multivariate binary logistic regression analyses to identify factors associated with depression symptoms. Statistical significance was declared at *P* < 0.05.

**Result:**

The prevalence of depression symptoms was found to be 53.9% (95% CI: 50.2, 57.0). In the multivariable logistic regression, female sex (AOR = 2.04, 95% CI: 1.35, 3.09), being single (AOR = 3.65, 95% CI: 3.48, 2.10, 5.78), widowed (AOR = 2.82, 95% CI: 1.27, 6.30), unable to read and write (AOR = 2.71, 95% CI: 1.14, 6.47), admission at medical ward (AOR = 1.59, 95% CI: 1.02, 2.46), history of mental illness (AOR = 1.59, 95% CI: 1.02, 2.46), and poor social support (AOR = 2.82, 95% CI: 1.57, 5.11) were factors significantly associated with depression symptoms.

**Conclusion:**

The prevalence of depression symptoms among admitted patients was high. Female sex, being single, widowed, unable to read and write, admission at medical ward, history of mental illness, and poor social support were factors significantly associated with depression symptoms. It is better for the Ministry of Health to give training on how to screen depression among medical and surgical patients, and interventions that would be addressing the awareness of the above factors would be beneficial to prevent further complications.

## 1. Introduction

Depression is a common mental illness globally characterized by sadness and loss of interest in activities that are normally enjoyable, accompanied by an inability to carry out daily functions [1, 2]. According to the World Health Organization report, more than three hundred twenty-two million people are living with depression in the world [3]. And it is a leading cause of disability and ill health globally [2–5]. It is the third leading cause of disease burden, and it accounts for 4.3% of total disability [6]. Lifetime and one-year prevalence of depression are approximately 13% and 5-7%, respectively [5, 7].

In 2017, the WHO report showed that the prevalence of depression ranged from 9 to 27% across different regions [3, 8]. In sub-Sahara, the magnitude of depression ranges from 15 to 30%, and in Ethiopia, it accounts for 9.1% [9, 10]. It is more common in females than males [1, 2, 9]. It contributes to its huge public health burden, including the impact of depression which increased the risk of suicide and premature mortality from cooccurring physical disorders [5, 11].

The WHO 2014 report revealed that the rate of depression among patients with physical illness was high, and it was two to three times more common in people with physical problems [3, 12]. Depression has a high prevalence among adult patients in clinical settings, which ranges from 5 to 30% [13, 14]. But it was high among admitted chronic medical patients [15]. Two meta-analyses were done across different countries among adult admitted individuals; the prevalence of depression was 15 to 60% and 9.6% to 16.5%, respectively [16, 17].

Several factors could play a role in the development of depression secondary to medical and surgical illnesses. These include repeated hospital admission history, poor social support, long hospital stay, previous history of mental illness, female sex, and admission in surgical or medical ward [18–22]. The comorbidity of depression with admitted medical and surgical patients negatively impacts the quality of life, functioning, adherence to medication, and high risk of suicide and increased morbidity and mortality [23, 24].

The vast majority of people with depression symptoms do not receive any care in many countries. Even in developed countries, nearly half of people with depression do not get treatment [2, 4]. The prevalence of depression is increasing, particularly in low- and middle-income countries including our country [6, 13].

However, research into depression symptoms and their determinants among people with medical and surgical patients in low- and middle-income countries is limited. Therefore, the aim of this study was to identify the prevalence of depression symptoms and associated factors among people with medical and surgical patients in Ethiopia which has a pivotal role in further intervention.

## 2. Methods and Materials

### 2.1. Study Setting and Period

An institution-based cross-sectional study was conducted from May to June 2019 at St. Paul's Hospital Millennium Medical College, Addis Ababa, Ethiopia. The medical school opened in 2007, but the hospital was established in 1968 by the late Emperor Haile Selassie. The hospital has both inpatient and outpatient services with 400 beds, and 2682 patients are admitted to the wards each month, especially 884 patients from the medical ward and 379 from the surgical ward. So, a total of 1263 participants were admitted to the medical and surgical wards per month.

### 2.2. Study Population

All admitted adult patients aged ≥18 were included, while patients found to be unconscious and unable to take consent were excluded.

### 2.3. Sample Size Calculation and Sampling Procedure

The sample size was calculated by using the single population proportion formula with 95% confidence interval (CI) and a 4% margin of error and taking the prevalence of depression 38% from a previously published study in our country [25]. This yields a total of 566 samples. Having assumed a 10% nonrespondent rate, the final sample size was 566 + 57 = 623 admitted medical and surgical patients recruited randomly by using a systematic sampling technique. The sampling interval was determined by dividing the total study participants who were admitted in medical and surgical wards during the month of data collection by the total sample size. Proportionally, 436 and 187 admitted patients were taken from medical and surgical wards, respectively; so, the selection skip interval for both wards was two. The first individual was selected by a lottery method from the admission register.

### 2.4. Data Collection

Data were collected using a pretested interviewer-administered questionnaire which contained depression as the dependent variable and several other determinant variables, including sociodemographic factors, clinical factors, social support, and substance use.

#### 2.4.1. Instruments

Social support was assessed by using the Oslo 3-item social support scale and used several studies. It provides a brief measure of social support and functioning and is considered to be one of the best predictors of mental health. It covered different levels of social support by measuring the number of people the respondents feel close to, the interest and concerns showed by others, and the ease of obtaining practical help from others. The sum score scale ranged from 3 to 14 and had three broad categories: “poor social support” 3-8, “moderate support” 9-11, and “strong support” 12-14, respectively [26]. The internal consistency of the Oslo social support scale in this study was 0.83. It has been used in Ethiopia in different clinical settings [22, 27, 28]. Substance use was assessed by using the WHO Alcohol, Smoking, and Substance Involvement Screening Test (ASSIST) guidelines for use in primary care. It is a brief screening questionnaire to find out people's use of psychoactive substance. It provides information about the substances people have ever used in their lifetime and at least one of any specific substances for nonmedical purpose in the past three months, respectively. Its Cronbach' alpha is 80%, sensitivity 0.80%, and specificity 71%, respectively [29]. Income was assessed by using the WHO poverty line which is less than or equal to 1.9 dollars per day as extreme poverty and greater than 1.9 dollars per day as the poverty line which is 1627 according to Ethiopian Birr during the study period [30].

Depression was assessed by using HADS (hospital anxiety and depression scale) which has 7-item questionnaire subscales for depression symptoms. Possible scores range from 0 to 21 for depression with cutoff points of greater than or equal to eight. In Ethiopia, it was validated, and its internal consistency was 0.76 for depression subscales [31].

### 2.5. Data Processing and Analysis

Data were entered into Epi-data software version 3.1 and imported to SPSS version 20 for analysis. Univariate, bivariate, and multivariate logistic regression analyses were done to see the association of each independent variable with the dependent variable. The strength of associations was evaluated using the adjusted odds ratio with a 95% CI, and a *P* value of less than 0.05 was considered statistically significant.

### 2.6. Ethical Considerations

Ethical approval was obtained from the Institutional Review Board (IRB) of the University of Gondar. A letter of permission was issued by Amanuel Mental Specialized Hospital. We received informed written consent from study participants. Confidentiality was maintained by omitting personal identifiers.

## 3. Results

### 3.1. Sociodemographic Characteristics

Out of a total of 623 participants, 590 completed the survey with a response rate of 94.7%. The mean age of the participants was 39.71 (±14.94) years, and above half (51%) of the participants were male. Nearly three-fifths (58.5%) were from urban residents. Out of the participants, 329 (55.8%) were Orthodox Christian followers, and 276 (46.8%) were married. Almost half of the study participants (46.8%) were living with their husband or wife, 219 (37.1%) were unable to read and write, 145 (24.6%) were farmers, and more than three-fifths (63.9%) have gotten ≤1627 Ethiopian Birr per monthly income (Table 1).

### 3.2. Clinical Characteristics of Respondents

Of the 590 participants, 410 (69.5%) were admitted to medical wards, one-third 31.4% was diagnosed with endocrine disorder, and two-fifths (42.5%) of them were staying in the hospital for a week. Almost two-fifths (63.1%) of the participants had a previous history of admission, 415 (70.3%) of the participants had multiple medical diagnoses, and about 63 (10.7%) of the respondents had a history of suicidal attempts (Table 2).

### 3.3. Social and Substance-Related Factors

Among the total of the participants, two-fifths 239 (40.5%) had poor social support. Three-fourths (76.8%) of the respondents had a history of lifetime substance use, 404 (68.5%) have been drinking alcohol, 88 (14.9%) were smoking cigarettes, and nearly 33.9% were chewing khat throughout their lifetime, respectively. At the moment, 26.1% of respondents have a history of current substance use (Table 3).

### 3.4. Prevalence of Depression among Respondents

The prevalence of depression symptoms among study participants was 53.9% with a 95% CI (50.20, 57.00). Over three-fifths (61.9%) were females, and more than two-fifths (46.2%) were male.

#### 3.4.1. Factors Associated with Depression Symptoms

Of the independent variables, female sex, being single, being widowed/r, unable to read and write, daily labor, living alone, admitted to medical ward, history of previous admission, history of mental illness, and poor social support yielded *P* value less than 0.2 in the bivariate logistic regression and were considered in the multivariate logistic regression model. The multivariate analysis suggested that female sex are two times more likely to develop depression symptoms compared to male sex (AOR = 2.04, 95% CI: 1.35, 3.09). Being single was over three times to develop depression symptoms compared to their counterparts (AOR = 3.65, 95% CI: 3.48, 2.10, 5.78), and widower/separated was 2.8 times more likely to be risky to depression when compared to married (AOR = 2.82, 95% CI: 1.27, 6.30). The odds of developing depression symptoms were 2.7 times higher among those unable to read and write when compared with college or university graduates (AOR = 2.71, 95% CI: 1.14, 6.47). The odds of developing depression were more than 1.5 times more likely among patients admitted in medical wards as compared with those who are admitted in surgical wards (AOR = 1.59, 95% CI: 1.02, 2.46). History of mental illness has 1.79 times increased the risk of depression compared to patients who had no history of mental illness (AOR = 1.79, 95% CI: 1.06, 3.05), and respondents who had poor social support were 2.82 times more likely to lead to depression as compared to respondents who had strong social support (AOR = 2.82, 95% CI: 1.57, 5.11) (Table 4).

## 4. Discussion

In the current study, the prevalence of depression symptoms and their possible association with various factors were assessed. The results revealed that a high proportion of depression symptoms were found among people admitted in medical and surgical wards. The prevalence of depression symptoms among people admitted in medical and surgical wards was found to be 53.9%.

Regarding prevalence, our result is in line with those of other two studies carried out in Northwest and Eastern Ethiopia which reported that the prevalence of depression symptoms was 54.6% and 57.9%, respectively [32, 33]. However, this finding is lower than those of studies conducted in India 60.5% [17] and Iran 58.8% [19]. The possible reason for the variation of the above prevalence might be due to the discrepancy of instruments, types of diagnosis and site of admission, study population contrasts, and the sociocultural difference between Ethiopia and the other countries.

On the other hand, our finding is higher than those of studies done in Ethiopia 38% [25], Nigeria 45.3% [34], Iran 42.3% [21], Italy 21% [17], England 38% [35], Jamaica 33.3% [36], and Brazil 28% [37]. The variation in the above rates might be due to differences in the sample sizes, age of participants, types of study design, the use of various scales and rating for assessing the level of depression symptoms, methodologies, types of admission site, types of patients, and sociocultural contrasts between Ethiopia and other countries.

There were different factors associated with depression symptoms in this study; female gender is two times more likely risky for depression symptoms when compared to male sex among patients admitted to medical and surgical wards. In this finding, 30% of females had depression which was higher than 23.6% of males. This is supported by studies done in Brazil among admitted medical patients; female sex had more than one and a half times of depression symptoms than male sex [38], and in Iran, female gender is significantly associated with depression symptoms among surgical patients compared with male sex [19]. Females are nearly twice as likely as men to be vulnerable to depression symptoms. Some mood changes and depressed feelings occur with normal hormonal changes. There are different factors that may increase the risk of depression symptoms in females such as biological factors, personal circumstances, and different countries with various sociocultural backgrounds. For example, in Ethiopia, female sex experiences some form of trauma during their lives like gender violence, discrimination, and mistreatment of women associated with medical or surgical illness leading to high stress, social withdrawal, and low self-esteem which in turn increased depression symptoms.

Being single was over three times likely to have depression symptoms compared to their counterpart, and widowed was 2.8 times more likely risky to depression when compared to married. This is consistent with other studies carried out in Uganda [39], China [40], and England [35]. Being widowed was associated with psychological distress among adults admitted to medical and surgical wards [39], living alone without their family members was highly associated among admitted patients [39], and being widowed has increased depression symptoms among admitted patients [40].

This study revealed that unable to read and write has nearly three times risk of depression symptoms compared to their counterparts, which is in line with other studies done in Iran; a lower educational level was significantly associated with depression symptoms among surgical patients [19], and in Brazil, a lower level of education has more than three times risk of depression among individuals admitted in a general hospital [41].

Adult patients admitted in medical wards are more than 1.5 times more likely to have depression as compared with those who are admitted in the surgical and medical wards. Depression symptoms are a common condition among medical patients, and its symptoms lead to increased morbidity and mortality rates, psychosocial functioning, and increased use of medical resources. But different studies revealed that depression symptoms are highly associated with admission in the surgical ward than the medical ward [32, 33, 42]. So, it needs further research, why patients admitted in medical wards are highly risky for depression symptoms compared to surgical ward patients.

This study showed that depression symptoms were significantly associated with prior history of mental illness. History of mental illness has 1.79 times increased the risk of depression symptoms compared to patients who had no history of mental illness. This was supported by a study done in Iran; a previous history of mental illness was a risk factor for depression symptoms in surgical patients [19].

Poor social support was nearly three times more likely to have depression symptoms compared to those who have good social support in this study that was comparable with the studies done in Ethiopia; poor social support has two times more likely risk to develop depression compared with good social support [27]; in another study in Ethiopia, poor social support has more than three times increased depression symptoms among medical patients [22], and in Wuhan, China, the absence of medical insurance and poor family support were highly associated with depression symptoms among admitted patients [40], and in Iran, lack of family support was a risk factor for depression disorders [19]. The present study has several strengths. Firstly, we used standardized instruments to measure depression symptoms; secondly, we adjusted the final multivariate model for the most important factors affecting depression. This study also has limitations; it was a cross-sectional study and thus cannot establish a causal link between associated factors and depression symptoms among admitted medical and surgical patients. The finding is likely only to hint at the complex interactions between depression symptoms and explanatory variables. So, the interpretation and usage of the study must be considered a limitation.

## 5. Conclusion

The prevalence of depression symptoms among admitted patients in medical and surgical wards was found to be 53.9%. Depression was significantly associated with female sex, being single, widowed, unable to read and write, site of admission, history of mental illness, and poor social support. It is better for the Ministry of Health to give training on how to screen depression among medical and surgical patients, and interventions that would address the awareness of the above factors would benefit the prevention of further complications. Further research on risk factors for depression symptoms should be conducted to strengthen and broaden this research.

## Figures and Tables

**Table 1:** Distributions of sociodemographics of participants admitted in medical and surgical wards at SPHMMC, 2019 (*n* = 590).

Variables	Category	Frequency	Percent
Age	Mean and SD	39.71 (±14.94)	

Sex	Male	301	51
	Female	289	49

Residence	Urban	345	58.5
	Rural	245	41.5

Marital status	Married	276	46.8
	Single	231	39.2
	Divorced	39	6.6
	Widowed/separated	44	7.5

Ethnicity	Amhara	248	42.0
	Oromo	254	43.1
	Gurage	58	9.8
	Others^∗^	30	5.1

Religion	Orthodox	329	55.8
	Protestant	126	21.4
	Muslim	105	17.8
	Catholic and others	30	5.1

Educational level	Unable to read and write	219	37.1
	Can read and write only	92	15.6
	Primary school	152	25.8
	Secondary school	84	14.2
	Above diploma	43	7.3

Jobs	Farmer	145	24.6
	Housewife	114	19.3
	Merchant	35	5.9
	Governmental employer	62	10.5
	Private	111	18.8
	Student	36	6.1
	Daily worker	87	14.7

Living condition	With husband or wife	276	46.8
	With family	100	16
	With relatives	35	5.9
	Alone	145	24.6
	With others^∗∗^	34	5.8

Monthly income	≤1627	377	63.9
	>1627	213	36.1

Others^∗∗^: with their children; others^∗^: Tigre, Wolayta, and Silte.

**Table 2:** Distribution of clinical factors of respondents admitted at medical and surgical wards at SPHMMC, Addis Ababa, Ethiopia, 2019 (*n* = 590).

Variables	Categories	Frequency	Percent
Site admission	Medical ward	410	69.5
	Surgical ward	180	30.5

Current diagnosis	Musculoskeletal d/r	41	6.9
	Genitourinary d/r	56	9.5
	Endocrine d/r	185	31.4
	Gastrointestinal d/r	103	17.5
	Infectious diseases	155	26.3
	Cardiovascular d/r	50	8.5

Duration of hospital stay	One week	251	42.5
	Two weeks	242	41.0
	Three weeks	41	6.9
	≥one month	56	9.5

Pervious history of admission	No	372	63.1
	Yes	218	36.9

Multiple diagnoses of medical illness	No	41	70.3
	Yes	17	29.7

History of suicidal attempt	No	527	89.3
	Yes	63	10.7

Feeling of pain	No	285	48.3
	Yes	305	51.7

Fear of death	No	488	82.9
	Yes	102	17.3

Fear of complication	No	248	42.0
	Yes	342	58

History of mental illness	No	46	78.8
	Yes	125	21.2

Family history of mental illness	No	480	81.4
	Yes	11	18.6

Family history of chronic medical illness	Yes	89	15.1
	No	501	84.9

**Table 3:** Psychosocial and substance use characteristics of respondents admitted at medical and surgical wards of SPHMMC, Addis Ababa, Ethiopia, 2019 (*n* = 590).

Variables	Categories	Frequency	Percent
Social support	Poor	239	40.5
	Moderate	266	45
	Good	85	14

Ever substance use	Yes	453	76.8
	No	137	23.2

Ever khat use	Yes	200	33.9
	No	390	66.1

Ever alcohol use	Yes	404	68.5
	No	186	31.5

Ever cigarette use	Yes	88	14.9
	No	502	85.1

Ever other substance use	Yes	22	3.7
	No	568	96.3

Current addictive substance use	Yes	175	29.7
	No	415	70.3

Current khat use	Yes	46	7.8
	No	544	92.2

Current alcohol use	Yes	154	26.1
	No	436	73.9

Current other addictive substance use	Yes	10	1.7
	No	580	98.3

**Table 4:** Bivariate and multivariate analyses among admitted patients in medical and surgical wards at SPHMMC, Addis Ababa, Ethiopia (*n* = 590).

Variable	Depression		COR (95% CI)	AOR (95%)
	Yes	No		
Sex				
Male	139	162	1	1
Female	179	110	1.89 (1.37, 2.63)	2.04 (1.35, 3.08)^∗^

Marital status				
Married	107	169	1	1
Single	159	72	3.49 (2.41, 5.04)	3.48 (2.09, 5.77)^∗∗^
Divorced	25	14	2.82 (1.40, 5.67)	2.28 (0.96, 5.42)
Widowed/separated	27	17	2.51 (1.31, 4.82)	2.82 (1.26, 6.29)^∗^

Educational level				
Unable to read and write	149	70	2.97 (1.51, 5.77)	2.71 (1.14, 6.45)^∗^
Can read and write only	51	41	1.73 (0.83, 3.59)	2.28 (0.90, 5.79)
Primary school	62	90	0.97 (0.48, 1.90)	1.06 (0.44, 2.56)
Secondary	38	46	1.15 (0.55, 2.41)	1.27 (0.50, 3.23)
College/university	18	25	1	1

Living conditions				
Wife/husband	127	149	1	1
Family	46	54	0.99 (0.63, 1.58)	0.78 (0.45, 1.35)
Relatives	20	15	1.56 (0.77, 3.18)	0.71 (0.29, 1.68)
Alone	106	39	3.19 (2.06, 4.94)	1.46 (0.80, 2.67)
Others^∗^	19	15	1.49 (0.73, 3.04)	0.65 (0.28, 1.54)

Site admission				
Medical ward	233	177	1.47 (1.04, 2.09)	1.59 (1.02, 2.47)^∗^
Surgical ward	85	95	1	1

Previous history of admission				
Yes	144	74	2.21 (1.57, 3.13)	1.42 (0.94, 2.16)
No	174	198	1	1

History of mental illness				
Yes	92	33	2.95 (1.90, 4.57)	1.79 (1.06, 3.05)^∗^
No	226	239	1	1

Social support				
Poor	166	73	2.68 (1.61, 4.46)	2.82 (1.56, 5.11)^∗^
Moderate	113	153	0.87 (0.53, 1.42)	0.86 (0.49, 1.51)
Good	39	46	1	1

Note: ^∗∗^<0.001; ^∗^<0.05. Others^∗∗^: with their children; Hosmer and Lemeshow Test = 0.52.

## Data Availability

The dataset during and/or analyzed during the current study is available from the corresponding author on reasonable request.

## Abbreviations

AMSH: Amanuel Mental Specialized Hospital
AOR: Adjusted odds ratio
CI: Confidence interval
HADS: Hospital anxiety and depression scale
OR: Odds ratio
PHQ-9: Patient health questionnaire-9
SPHMMC: Saint Paul's Hospital Millennium Medical College
SPSS: Statistical Package for the Social Sciences
UoG: University of Gondar
WHO: World Health Organization.
